# Malignant Transformation of Postmenopausal Endometriosis: A Systematic Review of the Literature

**DOI:** 10.3390/cancers13164026

**Published:** 2021-08-10

**Authors:** Luca Giannella, Chiara Marconi, Jacopo Di Giuseppe, Giovanni Delli Carpini, Mariasole Fichera, Camilla Grelloni, Lucia Giuliani, Michele Montanari, Salvatore Insinga, Andrea Ciavattini

**Affiliations:** Woman’s Health Sciences Department, Gynecologic Section, Polytechnic University of Marche, Via Filippo Corridoni, 16, 60123 Ancona, Italy; lucazeta1976@libero.it (L.G.); kiafosk@gmail.com (C.M.); jacopodigiuseppe@gmail.com (J.D.G.); giovdellicarpini@gmail.com (G.D.C.); fichera.mariasole@gmail.com (M.F.); camygrello@googlemail.com (C.G.); lucia-giuliani@alice.it (L.G.); michi.monta@gmail.com (M.M.); salvo.insinga@libero.it (S.I.)

**Keywords:** endometriosis, cancer, menopause, malignant transformation, hormone replacement therapy, surgery, years

## Abstract

**Simple Summary:**

Endometriosis is a disease that affects 6–10% of women of reproductive age. Although it is a benign condition, endometriotic lesions have around a 1% risk of malignant transformation. Since endometriosis is an estrogen-dependent disease, it can be expected to resolve or disappear in menopause. However, the prevalence of menopausal endometriosis is 2–4%. In these cases, the risk of malignant transformation is a rare but possible event. Data on the risk of these occurrences are scarce and of low quality. The most studied risk factors related to malignant transformation were hormone replacement therapy and the use of tamoxifen. However, the results were inconclusive. Furthermore, the impact of previous endometriosis and related surgery is unclear. Thus, we felt the need for an updated review on this topic focusing on the distribution of recurrent clinical conditions over a period of 50 years. In this regard, a systematic literature review on the malignant transformation of endometriosis-related lesions in postmenopause was performed to provide useful clinical information about patient characteristics, HRT use, and outcomes.

**Abstract:**

**Objective:** This study aimed to systematically review the existing literature on malignant transformation of postmenopausal endometriosis to provide information about patient characteristics, hormonal replacement therapy (HRT) use, and outcomes over a period of 52 years (1969–2021). **Methods:** According to PRISMA guidelines, we searched for (endometriosis OR endometriotic) AND (cancer OR malignancy OR malignant transformation) AND (menopause OR menopausal OR postmenopause OR postmenopausal) in Pubmed (all fields) (accessed on 12 February 2021) and Scopus (Title/Abstract/Keywords) (accessed on 12 February 2021) databases. The only filter used was the English language. Relevant articles were obtained in full-text format and screened for additional references. Eligibility/inclusion criteria: studies including full case description of malignant transformation of endometriosis-related lesions in postmenopause. **Results:** 75 studies, including 90 cases, were retrieved. The mean age was 55.8 ± 8.5 years. Overall, about 65% of women had a positive personal history of endometriosis/adenomyosis, and 64% of women underwent previous hysterectomy ± bilateral salpingo-oophorectomy. Forty-nine of 74 women used HRT (66.2%). Among the women who used HRT, estrogen-only treatment was taken by approximately 75%. Duration of HRT was longer than five years in 63.3% of cases. About 70% of subjects had histology of endometrioid adenocarcinoma or clear cell carcinoma. Follow-up outcome, available for 61 women, showed a survival rate of 78.7%, recurrence of 9.8%, death of 11.5%. The duration of follow-up had a median of 12 months (interquartile range, 6.75–25 months). Interestingly, over the years of case publication there was a significant inverse correlation with previous history of endometriosis (r = −0.28, *p* = 0.007), HRT use (r = −0.31, *p* = 0.006), and previous definitive surgery (r = −0.42, *p* < 0.001). **Conclusions:** In the malignant transformation of postmenopausal endometriosis, there are some recurrent clinical conditions: previous endometriosis, major definitive surgery before menopause, and estrogen-only HRT for a relatively long time. However, these clinical conditions have shown a drastic decrease over time. This could likely be the consequence of different attitudes and management of gynecologists linked to up-to-date scientific evidence about the use of major surgery in gynecological pathologies. Malignant transformation of postmenopausal endometriosis is a clinical challenge to be explored further.

## 1. Introduction

Endometriosis is an estrogen-dependent complex clinical syndrome characterized by the presence of ectopic endometrial-like tissue [1]. It is a pathological clinical condition that mainly affects women of reproductive age [1]. Various theories have been proposed to explain it: retrograde menstruation, the local immune alterations, imbalance between pro- and anti-angiogenic factors [1]. A multifactorial process is likely to occur in the etiopathogenesis of endometriosis. In women of reproductive age, it often causes infertility and chronic pelvic pain leading to severe functional limitations [2].

Another aspect of interest related to endometriosis is the risk of malignant transformation. A recent metanalysis showed that: (1) the risk of ovarian cancer doubles in patients with endometriosis; (2) conversely, the risk of endometrial and cervical cancer does not appear to be higher in these women [3]. Furthermore, when an endometriotic lesion turns into cancer, it appears to have a better prognosis. In a large metanalysis, endometriosis-associated ovarian cancers showed favorable characteristics, including early-stage disease, low-grade disease, and specific histology such as endometrioid or clear cell carcinoma [4].

After the reproductive period, endometriosis is thought to resolve or remain in a state of inactivity. Although the hypoestrogenic state related to menopause may suggest it, postmenopausal endometriosis can affect up to 4% of women [5]. Recurrences or malignant transformations are rare but possible events. Data on the risk of these occurrences are scarce and of low quality [5,6]. The most studied risk factors related to malignant transformation were hormone replacement therapy (HRT) and the use of tamoxifen for breast cancer [5,6]. However, the results were inconclusive. Moreover, the impact of previous endometriosis and related surgery is unclear. It is, therefore, necessary to raise awareness that endometriosis is not exclusively a condition of the reproductive phase.

The purpose of this study was to systematically review the existing literature on malignant transformation of postmenopausal endometriosis to provide information about patient characteristics, hormonal replacement therapy (HRT) use, and outcomes over a period of 50 years.

## 2. Materials and Methods

The PRISMA (Preferred Reporting Items for Systematic Reviews and Meta-Analyses) guidelines were followed to systematically review the literature by searching the Pubmed and Scopus databases [7]. The present study assessed the following PICOS (Population, Intervention, Comparison, Outcomes) questions:

- Population: postmenopausal women with malignant endometriosis transformation;
- Intervention: personal history including data on previous endometriosis and surgery, hormone replacement therapy (HRT) use, and duration over the years of case publication;
- Comparison: no comparison expected;
- Outcomes: (1) presence of common patient characteristics based on descriptive analysis, (2) distribution of recurrent clinical conditions over time, (3) follow-up outcomes when available (alive, recurrence, death).

Study design: retrospective observational studies (case reports, case series). Eligibility/inclusion criteria: studies including full case description of malignant transformation of endometriosis-related lesions in postmenopause (including spontaneous and/or surgical menopause). In this regard, the malignant transformation of endometriotic foci had to comply with Sampson’s and Scott’s criteria [8,9]. Exclusion criteria: review articles were excluded; research articles on endometriosis and risk of malignancy including postmenopausal status as simple independent variables (without a full case description); cases in a non-English language.

Information sources and search strategy: we searched for (endometriosis OR endometriotic) AND (cancer OR malignancy OR malignant transformation) AND (menopause OR menopausal OR postmenopause OR postmenopausal) in Pubmed (all fields) (accessed on 12 February 2021) and Scopus (Title/Abstract/Keywords) (accessed on 12 February 2021) databases. The only filter used was the English language. Relevant articles were obtained in full-text format and screened for additional references.

Study selection: three independent reviewers (Chiara Marconi, Camilla Grelloni, Mariasole Fichera) selected the studies using a 2-steps screening method. At first, the screening of titles and abstracts was performed to assess eligibility and inclusion criteria and exclude irrelevant studies. Afterward, the three reviewers evaluated full texts of included articles to (1) assess study eligibility and inclusion criteria and (2) avoid duplications of the included cases. Three other authors (Lucia Giuliani, Michele Montanari, Salvatore Insinga) manually searched reference lists to search for additional relevant publications. Jacopo Di Giuseppe and Giovanni Delli Carpini checked the data extracted.

The objective of this systematic review was: (1) to provide and summarize the literature on a rare event on which there is scant data, such as the malignant transformation of endometriosis-related lesions in postmenopause; (2) to provide any information about patient characteristics, HRT use, and outcomes over a period of 52 years (1969–2021).

Data collection process/data items: data collection was study-related (authors and year of study publication) and case-related (patient characteristics, signs/symptoms at clinical presentation, hormone replacement therapy data, cancer lesion characteristics, treatment, and outcome).

Statistical analysis: the collected data were reported as continuous or categorical variables. Continuous variables were tested for normal or not-normal distribution using the Kolmogorov-Smirnov test. According to distribution, they were expressed as mean ± standard deviation or median and interquartile range. Categorical variables were expressed as frequency and percentage. Correlation analysis was used to determine whether the values of two variables were associated (if they had normal distribution). When the distribution of variables was not normal, the degree of relationship between the variables was determined using Rank correlation (the Spearman’s coefficient). MedCalc^®^ Statistical Software version 20 (MedCalc Software Ltd., Ostend, Belgium; https://www.medcalc.org; 2021) was used.

## 3. Results

### 3.1. Literature Review Details

**Figure 1:**
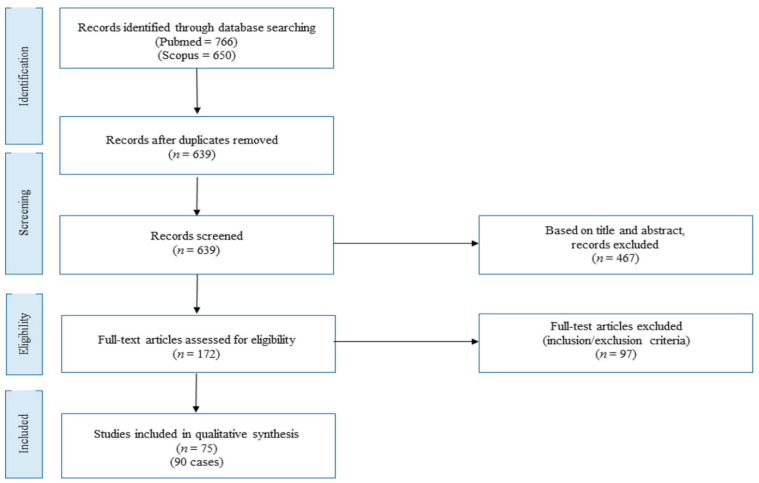
Literature review flow-chart.

Figure 1 shows the literature review flowchart. We retrieved 766 articles on Pubmed and 650 papers on Scopus databases (accessed on 12 February 2021). After duplicates were removed, 639 articles were assessed. Based on title and abstract, 467 records were excluded. Then, the full text of 172 papers was evaluated for eligibility. Based on inclusion and exclusion criteria, 97 articles were further removed. Finally, 75 studies were assessed for qualitative synthesis, including 90 cases (Figure 1). All studies and patients are detailed in Supplementary Table S1 [10,11,12,13,14,15,16,17,18,19,20,21,22,23,24,25,26,27,28,29,30,31,32,33,34,35,36,37,38,39,40,41,42,43,44,45,46,47,48,49,50,51,52,53,54,55,56,57,58,59,60,61,62,63,64,65,66,67,68,69,70,71,72,73,74,75,76,77,78,79,80,81,82,83,84].

### 3.2. Patient Characteristics

Table 1 presents patient characteristics. The mean age was 55.8 ± 8.5 years. Most of the women were parous (80%). Comorbidities, such as diabetes and hypertension, were present in 4.5% and 3.4% of women, respectively. Breast cancer was found in 7 women (7.8%), while leiomyomatosis was present in 19%. Obesity was present in 3 cases (3.4%), whereas past or current tamoxifen users were 6 (6.8%). About 65% of women had a positive personal history of adenomyosis and/or endometriosis. Six out of eighty-nine women (6.7%) underwent a hysterectomy, while fifty-one patients (57.3%) underwent a hysterectomy with bilateral salpingo-oophorectomy. The only salpingo-oophorectomy was performed in 52/89 women (58.4%). Endometriosis was the indication for previous surgery in 55.2% of cases.

### 3.3. Signs/Symptoms at Clinical Presentation

The most frequent sign at clinical presentation was pelvic mass (34.4%), followed by hematuria (8.9%), dyschezia (5.6%), weight loss (3.3%), and constipation (2.2), and 45.6% of women showed no sign (Table 2). Abnormal uterine bleeding was found in 43.3% of cases (Table 2).

The most frequent onset symptom was pelvic/abdominal pain (55.6%) (Table 2). There were also urinary symptoms and dyspareunia (2.2% and 1.1%, respectively) (Table 2). The women were asymptomatic in 41.1% of cases (Table 2).

Twenty-one women showed no signs and symptoms (23.3%). The majority of women (76.7%) presented signs or symptoms at clinical presentation (Table 2).

### 3.4. Hormone Replacement Therapy Data

Forty-nine of 74 women used HRT (66.2%) (Table 3). Among the women who used HRT, estrogen-only therapy was taken by approximately 75% of the women (Table 3). Duration of treatment had a median of 11 years. The course of treatment was longer than five years in most women (63.3%) (Table 3).

### 3.5. Cancer Lesion Characteristics

Table 4 shows the characteristics of the cancerous lesion. Approximately 70% of cases had histology of endometrioid adenocarcinoma or clear cell carcinoma (Table 4). The most frequent localization of the lesions was at the level of the pelvis (23.9%), ovary (18.2%), and vagina (17.0%) (Table 4). Most of the lesions had a single localization (64.4%) (Table 4).

### 3.6. Treatment and Outcome

Almost all women underwent surgical treatment (92.8%) (Table 5). Surgery included excision of the mass (25.3%), hysterectomy with bilateral salpingo-oophorectomy (24.1%), and surgical debulking (15.7%) (Table 5). Adjuvant medical treatment was performed in about 60% of cases, while neoadjuvant therapy was necessary only in one woman (Table 5). The outcome, available for 61 women, showed a survival rate of 78.7%, recurrence of 9.8%, and death of 11.5% (Table 5). The duration of follow-up had a median of 12 months (interquartile range, 6.75–25 months) (Table 5). The latest data was available for 45 women (Table 5).

According to the years of case publication, patients’ outcomes showed no significant differences (Table 6). However, it should be emphasized that after 2010 there was no death, but the recurrences increased compared to previous years (5.3%, 4.2%, 22.2%) (Table 6).

### 3.7. Distribution of Patient Characteristics over Time

**Figure 2:**
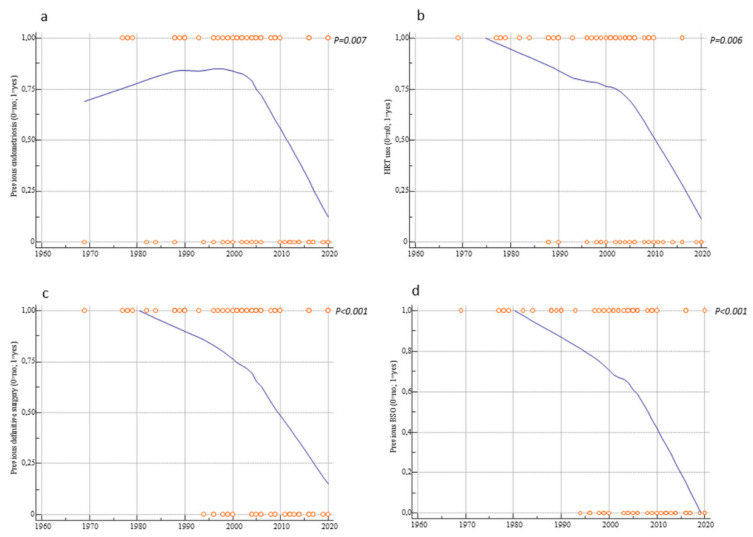
Scatter diagrams showing the trend over time of (**a**) previous endometriosis; (**b**) hormonal replacement therapy (HRT) use; (**c**) previous definitive surgery; (**d**) previous bilateral salpingo-oophorectomy (BSO).

**Figure 3:**
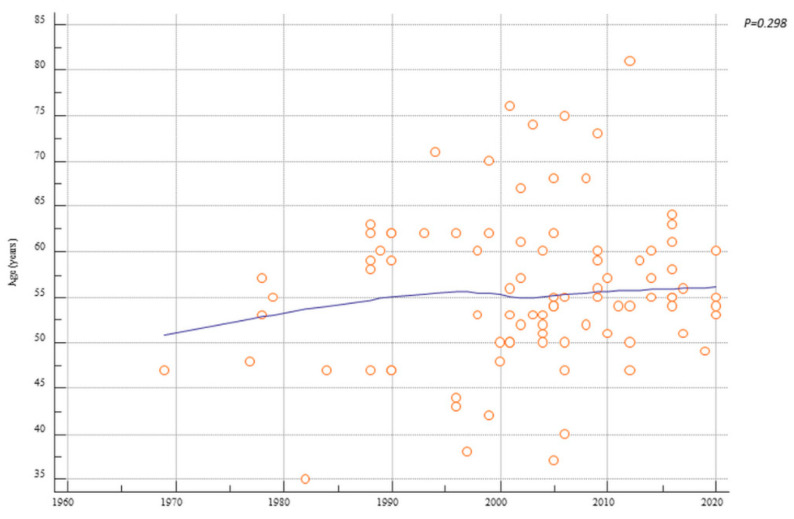
Scatter diagrams showing the trend over time of patients’ age affected by malignant transformation of postmenopausal endometriosis.

**Figure 4:**
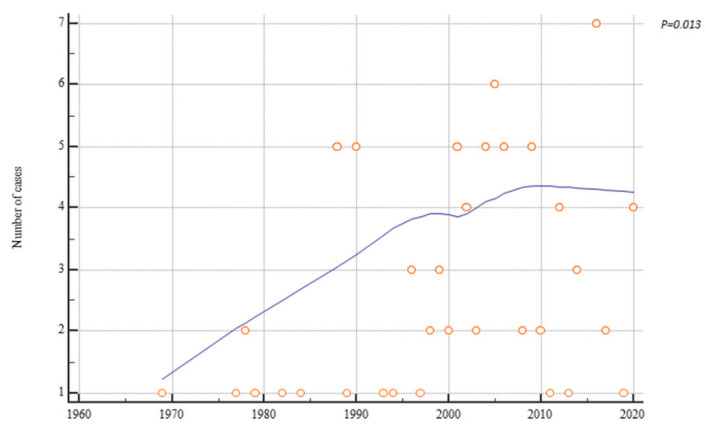
Scatter diagrams showing the trend over time of postmenopausal endometriosis transformation cases.

Interestingly, over a period of 52 years there was a significant inverse correlation with previous history of endometriosis (r = −0.28, *p* = 0.007), HRT use (r = −0.31, *p* = 0.006), previous definitive surgery (hysterectomy ± bilateral salpingo-oophorectomy) (r = −0.42, *p* < 0.001), and previous bilateral salpingo-oophorectomy (r = −0.49, *p* < 0.0001) (Figure 2). Patients’ age showed no correlation with the years of case publication (r = 0.11, *p* = 0.298) (Figure 3). Nevertheless, there was a slight increase in age over time (Figure 3). Finally, there was a significant positive trend over time of the number of cases of malignant transformation in postmenopausal endometriosis (r = 0.26, *p* = 0.013) (Figure 4).

### 3.8. Missing Data

In total, 90 women were included in this systematic review. Data on hypertension, diabetes, leiomyomatosis, body mass index and previous surgery were available in 89/90 cases (Table 1). HRT is specified in 74 women and is not reported in 16 patients (Table 3). The duration of HRT is determined in 44/49 cases (Table 3). Cancer localization is available in 88/90 women (Table 4). The surgical treatment is reported in 83/90 patients (Table 5). Data on medical treatment was available in 79 patients (Table 5). Follow-up outcomes are available for 61/90 women (Table 5). The exact duration of follow-up is available for 45 out of 90 women (Table 5).

## 4. Discussion

Endometriosis is a pathological clinical condition that mainly affects women of reproductive age. However, about 2–4% of postmenopausal women have symptomatic endometriosis [5]. Furthermore, this prevalence may be underestimated as other authors report higher rates [85,86]. Given the absence of menstruation and the hypoestrogenic state, the onset of endometriosis in menopause would suggest other pathophysiological mechanisms than those of the reproductive age. In addition, we do not know whether the lesions found in older women are recurrent/persistent diseases or new implants. The matter is subject to a discussion without clear and definitive evidence. Bulun et al. showed that endometriotic lesions themselves could produce estrogens through autocrine and paracrine effects [87]. This could explain the persistence or recurrence of lesions in a hypoestrogenic state such as menopause [88]. Other sources of estrogen production could play a role in postmenopausal endometriosis: obesity, phytoestrogen intake, HRT use, tamoxifen therapy [5]. In these latter cases, quiescent endometriotic foci in menopause may be stimulated exogenously through the treatments mentioned above [5].

Although endometriosis is a benign condition, it presents a risk of malignant transformation. Overall, it is reported that about 1% of ovarian endometriosis can turn into cancer [89,90]. However, a prospective study including approximately 6500 women with ovarian endometrioma and a mean follow-up of around 13 years showed a standardized incidence ratio of malignant transformation of 8.95 [91]. Regarding this data, cases of malignant transformation are likely to be negligible in pelvic pain or infertility practice. There may be a selection bias in hospital studies that have oncologists on staff. Cancers can be more common in the hospital, and endometriosis with only pelvic pain or infertility in the surgery center. Interestingly, the period in which the risk of malignant transformation increased was the perimenopausal one with medians between 45–49 years [91,92,93], hence the importance of paying close attention to endometriotic ovarian cysts in perimenopausal women.

Malignant transformation of postmenopausal endometriotic lesions is a rare occurrence. To date, there are no defined percentages about its prevalence. The data are obtained from studies, including case reports and case series. In a detailed systematic review, Gemmell et al. reported 25 cases of postmenopausal malignant transformation in women using HRT with a previous history of endometriosis [6]. Most of the patients were in surgical menopause (22/25, 88%). They showed that most women used estrogen-only HRT (19/25, 76%) with a median duration of approximately seven years (range 3–20 years). The most frequent signs and symptoms at clinical presentation included abdominal/pelvic pain, vaginal bleeding, and the presence of palpable masses. Histology was endometrioid adenocarcinoma in 72% of cases, and most patients underwent surgical treatment followed by adjuvant therapy. Follow-up outcomes were generally favorable, with a 12% mortality rate over a mean observation period of 19.4 months. Tan et al. reported 62 cases of malignant transformation of endometriosis in menopause [5]. The mean age was 58.2 years; half of the women had used HRT (71% with unopposed estrogen therapy); the mean duration of HRT was ten years; most of the lesions had endometrioid adenocarcinoma histology (67.7%).

To our knowledge, the present systematic review provides the most extensive and updated data on the topic. Overall, our descriptive analysis is in line with the results described above. Some recurrent clinical conditions emerged: the previous history of endometriosis/adenomyosis, definitive surgery before menopause (hysterectomy with salpingo-oophorectomy), and estrogen-only HRT use. Follow-up outcomes showed a favorable survival rate (about 80%) over a mean observation period of 12 months (range 6.75–25 months).

Based on these data, previous authors speculated that postmenopausal women with endometriosis malignant transformation likely had a prior history of severe endometriosis that led to definitive surgical treatment before menopause. This occurrence would explain the high percentage of women on estrogen-only HRT taken for relatively long periods. However, our results showed that 55% (37/67) of women undergoing surgery had endometriosis as an indication for intervention. This means that in 36% of cases (21/58 women with endometriosis), the diagnosis of endometriosis was likely an incidental finding on the final histological examination. In this regard, it is likely that the diagnostic work-up of endometriosis, when performed by generalist gynecologists, may fail to recognize endometriotic lesions both preoperatively and intraoperatively. In an interesting paper, Griffiths et al. reported that rectovaginal endometriosis often goes unrecognized during the first surgery [94].

Interestingly, our correlation analysis showed that these recurrent clinical conditions (history of endometriosis, major surgery, HRT use) decreased significantly over time (especially after 2010). Major surgery in asymptomatic affected women or benign conditions is likely to have decreased over the years based on new and updated recommendations [1,2,95,96]. This, in turn, could explain the negative trend over time of previous endometriosis (positive personal history may be missing among asymptomatic women in the absence of a histologic diagnosis) and long-term HRT. Overtreatment could likely have been much more frequent in the past [95,96]. This new analysis suggests that the recurrence of some clinical conditions may be due to a different attitude of gynecologists about the use of major surgery in some pathologies in the past years.

Although the selected cases may not represent the true prevalence of the disease, the data is worthy of mention. Interestingly, our analysis showed that the number of instances of postmenopausal malignant transformation increased over time. Conversely, we know that other studies showed a negative trend over the years in the incidence of endometriotic disease [97,98]. Although these data may seem contradictory, these current occurrences may be those cases of asymptomatic endometriosis that occurred 20–30 earlier. We will have to wait for the next few years to evaluate the next trend. A further increase of such cases would underline how these recurrent clinical conditions do not have a causal role in malignant transformation.

Further findings are worthy of discussion. The age trend increased over the years, albeit not significantly. It appears that women managed in past years had an earlier age when cancer occurred (Figure 3). Furthermore, deaths are absent after 2010 (Table 6). It may be an overextrapolation to state that past managements represent an adverse prognostic factor. Advances in oncology over the years could likely have a significant impact. Although the outcomes appear favorable, follow-up data are still too incomplete to provide adequate information on the prognosis.

A further emerging aspect of our data is the high percentage of previous bilateral salpingo-oophorectomy in women with subsequent malignant transformation. From our data, 58.4% of women underwent bilateral salpingo-oophorectomy procedures. These data seem to confirm further that the “risk-reducing surgical treatment” in women with previous endometriosis approaching menopause is not cost-effective [99]. On the contrary, the importance of removing all endometriotic lesions detected intraoperatively in any anatomical site is to be emphasized. These foci must be excised to obtain a histological diagnosis and reduce the future risk of recurrence/malignant transformation. Destructive treatment of minor endometriotic localizations is no longer acceptable [2].

The mechanism underlying this rare occurrence is unclear. It is likely that during surgical treatment, some not-macroscopically-visible endometriotic foci may be overlooked and left there. Furthermore, the same surgery can lead to an immediate inflammatory stimulus that favors the implantation and/or activation of any endometriotic foci not detected intraoperatively. When surgery is not performed, the presence of asymptomatic endometriosis locations may likely persist for a long time. These lesions may receive autocrine, paracrine, and exogenous stimuli along with cancer-predisposing gene mutations [99,100]. The use of estrogen-only HRT appeared to be recurrent in old cases. As a result, although solid evidence is missing, current recommendations on HRT include continuous combination formulations or Tibolone in women with previous endometriosis [2]. Furthermore, the choice of HRT use must balance the risk of bone and cardiovascular diseases with the possibility of recurrence/malignant transformation of endometriotic lesions [6]. To date, there is little data to provide solid evidence-based recommendations. There is a need for randomized controlled trials or extensive observational studies on this topic to give an accurate and individualized evaluation and information.

### Conclusions

In the malignant transformation of postmenopausal endometriosis, there are some recurrent patient characteristics:

- Previous endometriosis
- Definitive gynecological surgery before menopause
- Estrogen-only HRT for a relatively long time.

However, these clinical conditions have shown a drastic decrease in more recent years. It is likely that this could be the consequence of different attitudes and management of gynecologists linked to up-to-date scientific evidence about the use of major surgery in gynecological pathologies. Malignant transformation of postmenopausal endometriosis is a clinical challenge to be explored further.

## Figures and Tables

**Table 1:** Patient characteristics.

Variables	Sample Size (90) Available Data (%)
**Age at diagnosis** **(mean ± standard deviation)**	55.8 ± 8.5
**Nulligravid**	18/90 (20)
**Hypertension**	4/89 (4.5)
**Diabetes**	3/89 (3.4)
**Breast cancer**	7/90 (7.8)
**Leiomyomatosis**	17/89 (19.1)
**BMI ≥ 30**	3/89 (3.4)
**Tamoxifen use**	
Current use	2 (2.2)
Past use	4 (4.4)
No	84 (93.3)
**Previous endometriosis/adenomiosis**	
Adenomiosis	4 (4.4)
Endometriosis	44 (48.9)
Adenomiosis + Endometriosis	10 (11.1)
No	32 (35.6)
**Previous surgery**	
Hysterectomy	6/89 (6.7)
Hysterectomy + BSO	51/89 (57.3)
Other surgery	10/89 (11.2)
No surgery	22/89 (24.7)
**Previous BSO**	52/89 (58.4)
**Endometriosis as indication for previous surgery**	
Yes	37/67 (55.2)
No	30/67 (34.8)

BMI: body mass index; BSO: bilateral salpingo-oophorectomy.

**Table 2:** Signs/symptoms at clinical presentation.

Signs	Sample Size (90) Available Data (%)
Constipation	2 (2.2)
Dyschezia	5 (5.6)
Hematuria	8 (8.9)
Pelvic mass	31 (34.4)
Weight loss	3 (3.3)
None	41 (45.6)
Abnormal uterine bleeding	39/90 (43.3)
**Symptoms**	Sample size (90) Available data (%)
Dyspareunia	1 (1.1)
Urinary symptoms	2 (2.2)
Pelvic/abdominal pain	50 (55.6)
Asymptomatic	37 (41.1)
**No sign and symptom**	21/90 (23.3)

**Table 3:** Hormone replacement therapy data.

Hormone Replacement Therapy	Sample Size (90) Available Data (%)
**HRT use**	
Yes	49/74 (66.2)
No	25/74 (33.8)
**HRT type**	
CEE	8/49 (16.3)
E only	28/49 (57.1)
E + T	5/49 (10.2)
CT	7/49 (14.3)
Soy Isoflavones	1/49 (2.1)
**Duration of treatment (years)**	
Median (interquartile range)	11 (5–14)
≤5 years	16/44 (36.3)
>5 years	28/44 (63.7)

CEE: conjugated equine estrogen; E: estrogen; T: testosterone; CT: combined therapy; HRT: hormone replacement therapy.

**Table 4:** Cancer lesion characteristics.

Histology	Sample Size (90) Available Data (%)
EAC	45 (50.0)
CCC	9 (10.0)
EAC + CCC	6 (6.7)
Adenocarcinoma	16 (17.8)
Sarcoma	11 (12.2)
Other histology	3 (3.3)
**Cancer location**	Sample size (90) Available data (%)
Abdomen	4/88 (4.5)
Bladder	5/88 (5.7)
Cervix	3/88 (3.4)
Ovary	16/88 (18.2)
Parametrium	1/88 (1.1)
Pelvis	21/88 (23.9)
Rectum	8/88 (9.1)
Ureter	8/88 (9.1)
Uterus	7/88 (8.0)
Vaginal vault	15/88 (17.0)
**Involved site**	Sample size (90) Available data (%)
Multiple	32 (35.6)
Single	58 (64.4)

EAC: endometroid adenocarcinoma; CCC: clear cell carcinoma.

**Table 5:** Treatment and outcome.

Surgical Treatment	Sample Size (90) Available Data (%)
Debulking surgery	13/83 (15.7)
Mass excision	21/83 (25.3)
RAH + BSO	2/83 (2.4)
TH + BSO	20/83 (24.1)
Other surgery	21/83 (25.3)
No surgery	6/83 (7.2)
**Medical treatment**	Sample size (90) Available data (%)
Adjuvant CHT	30/79 (38.0)
Adjuvant CHT + RT	4/79 (5.1)
Adjuvant RT	11/79 (13.9)
Neoadjuvant CHT	1/79 (1.3)
No medical treatment	33/79 (41.8)
**Outcome**	Sample size (90) Available data (%)
Alive	48/61 (78.7)
Recurrence	6/61 (9.8)
Deceased	7/61 (11.5)
**Follow-up duration (months)**	45/90
Median (interquartile range)	12 (6.75–25)

RAH: radical abdominal hysterectomy; TH: total hysterectomy; BSO: bilateral salpingo-oophorectomy; CHT: chemotherapy; RT: radiotherapy.

**Table 6:** Patients’ outcome according to the years of case publication.

Outcome	Years of Case Publication			*p* Value
	<2000	2000–2009	≥2010	
	(19)	(24)	(18)	
	n (%)	n (%)	n (%)	0.102
Alive	14 (73.7)	20 (83.3)	14 (77.8)	
Deceased	4 (21.1)	3 (12.5)	0 (0.0)	
Recurrence	1 (5.3)	1 (4.2)	4 (22.2)	

## Data Availability

The data supporting the findings of this study are available within the article and its Supplementary Materials.

## Supplementary Materials

The following are available online at https://www.mdpi.com/article/10.3390/cancers13164026/s1, Table S1: Detailed data synthesis of included studies.
