# Supplementary material for: Malignant Transformation of Postmenopausal Endometriosis: A Systematic Review of the Literature
Source: Cancers (Basel). 2021 Aug 10;13(16):4026. doi: 10.3390/cancers13164026 (PMC8394809; doi:10.3390/cancers13164026)
Supplement: Supplementary file 1 [file cancers-13-04026-s001.zip › cancers-1331857-supplementary.pdf]

Supplementary Table 1. Detailed data synthesis of included studies.

Authors (Year)	Age	HRT use	Personal history	Past endometriosis/ adenomyosis	Past surgery	*Surgery indication: endometriosis*	Signs/Symptoms	Surgical treatment	Medical treatment	Locations	Histology	Follow-up (months)
**Carbone F. et al.[10] (2020)**	53	No	G0	Endometriosis	TAH and BSO	*Yes*	Abdominal pain	Debulking surgery	No	Left pelvic side wall	Low-grade sarcoma	4, alive
**Chih-Hsuan S. et al.[11] (2020)**	60	U	G0	No	None	*No*	Abdominal pain, weight loss. A bulky mass palpable at lower abdomen	Debulking surgery	CHT (taxane/ platium)	Adnexa	Right ovary: EAC Left ovary: CCC	U
**Cope AG. et al. [12] (2020)**	55	U	None relevant	Endometriosis, adenomyosis	Hysterectomy	*Yes*	Dyspareunia and AVB, pelvic cramping, and urinary urgency. Firm, fixed mass in the right upper vagina.	Surgery	No	Vaginal apex, right ovary, rectum	EAC	U
	54	No	None relevant	No	Hysterectomy; pelvic resection.	*No*	Pelvic pain and left ureteral obstruction	Pelvic mass surgical resection	CHT	Left pelvic sidewall, sigmoid colon, left ureter	EAC and CCC	120, recurrence
**Uehara T. et al.[13] (2019)**	49	No	Infertility treatment	No	None	*No*	AVB. Hard mass in the left cervix and parametrium	RAH, BSO and pelvic LNT	CHT	Cervical stroma, left parametrium	EAC	12, alive
**Chang C. et al.[14] (2017)**	56	U	G3 P3. Obesity, diabetes mellitus, hypercholesterolemia, and hypertension.	No	None	*No*	AVB and pelvic pain	TLH, BSO, omental and peritoneal biopsy	RT	Uterus, paratubal	EAC	U
**Tashima L. et al.[15] (2017)**	51	U	3G 3P	No	None	*No*	AVB, slight anemia	TAH and BSO	CHT	Uterus, ovary	EAC	12 alive
**Roncati L. et al.[16] (2016)**	64, 62	U	None relevant	No	None	*No*	AVB	TAH, BSO and loco-regional lymphadenectomy	No	Endometrium, ovaries	G2 EAC	U
**Pontrelli G. et al.[17] (2016)**	58	EP for 5 y	Fibromatosis	Endometriosis	AH and BSO	*No*	Severe and recurrent endometriosis	LPS with total colpectomy, parametrectomy and partial cystectomy	No	Vagina, left parametrium	Primary vaginal adenosarcoma	U
**Andriola V. et al.[18] (2016)**	54	No	G3 P0; BMI 29; fibromatosis	No	Cesarean section; TAH and BSO	*No*	Pelvic and anal pain, constipation, oliguria	Low rectal resection	No	Pelvis (between rectum and sacrum)	Well differentiated EAC	U
**Lu B. et al.[19] (2016)**	64	No	G2 P2. Diabete mellitus type II	No	None	*No*	Left low abdominal pain	TAH and BSO, omentectomy, pelvic LNT	CHT (taxol/ carboplatin)	Uterus, left adnexus	Adenocarcinoma serous carcinoma	1, alive
	55	U	G1. Multiple leyomiomas. Diabete mellitus type II, hyperthesion, asmha	Adenomyosis	Cesarean section	*No*	AVB	TAH, BSO, omentectomy and pelvic lymphoadenectomy	CHT (taxol/ carboplatin)	Uterus	Endometrial serous carcinoma	U, alive
	55	U	G2 P1	Adenomyosis	Cesaren section, STH	*Yes*	AVB	Radical resection of cervical stump, BSO, partial resection of rectum	No	Cervical stump	Endometrial serous carcinoma	44 alive
**Mohling S.I.[20] et al. (2016)**	61	E+T for 8-10 y	Hypothyroidism	Endometriosis	TAH and BSO	*Yes*	Left lower quadrant mass and pain, dyspareunia, hematuria	LPT with en bloc resection of the tumor	CHT (carboplatin/paclitaxel)	Pelvis, rectosigmoid colon	EAC	U alive
**Ijichi S. et al.[21] (2014)**	60	U	G3 P0	No	2 cesarian sections	*No*	Growing mass	Radical resection of the abdominal wall mass	No	Abdominal wall (cesarean scar)	CCC	8+15, recurrence
**Yu X.J. et al.[22] (2014)**	55	No	Dismenorrhea, fibromatosis	No	None	*No*	Right abdomen lower pain; weakness of limbs	TAH, BSO, omentectomy, right infundibulopelvic ligament resection	CHT (bleomicina/ etoposide/ cisplatin)	Right ovary	Mixed germ cell tumor and CCC	U, alive
**Taga S. et al.[23] (2014)**	57	U		No	None	*No*	AVB	TAH; BSO; pelvic limphadenectomy	CHT	Uterus	EAC	60, recurrence
**Lah K. et al.[24] (2013)**	59	U	Hypercholesterolemia	No	2 cesarean sections and tubal ligation	*No*	7-year history of intermittent hematuria	Robotic-assisted anterior pelvic exenteration and pelvic LNT	Neoadjuvant CHT (cisplatin/ gemcitabine) CHT (carboplatin/ paclitaxel	Bladder	CCC	12, alive
**Hansen K. et al.[25] (2012)**	81	U	Menopause at 50	No	None	*No*	AVB, abdominal fullness	TAH, BSO, omentectomy, pelvic and paraortic LNT	No	Right ovary, uterus	EAC	35, alive
**Shalin S. et al.[26] (2012)**	47	U	4G 4P	No	Previous caesarian section	*No*	Pelvic pain, tender subcutaneous nodule on abdomen with daily menstrual-like cramping	Wide resection of the abdominal wall mass, inguinal and iliac LNT	CHT; RT	Abdominal wall (near cesarean scar)	CCC	5+2, recurrence
**Mann S. et al.[27] (2012)**	54	No	None relevant	No	None	*No*	Gross hematuria, irregular menses	Transurethral resection of the mass; TAH, BSO, partial cystectomy, pelvic lymph node dissection and anterior vaginal wall resection	No	Bladder invading rectum	CCC	U
**Coyne J.D. et al.[28] (2012)**	50	U	Aangiomixoma treated with surgery	No	LPT with asportation of abdominal angiomixoma	*No*	Abdominal fullness; constipation	TAH and BSO, asportation of abdominal mass, washing	No	Abdominal wall, retroperitoneum, pelvis, uterus and upper vagina	Angiomixoma	U
**Takahashi Y. et al.[29] (2011)**	54	No	U	No	None	*No*	Abdominal distension	TAH, BSO, total omentectomy, appendocectpmy, pelvic and para aortic LNT	CHT (paclitaxel/ carboplatin; doxorubicina/paclitaxel)	Ovary; liver; paraortic lymphnodes	CCC with hepatoid carcinoma foci	48, alive

**Healy K.A. et al.[30] (2010)**	51	E since 38 y	End-stage renal disease for glomerular nephritis, menorrhagia	No	TAH and BSO. Left-renal transplant	*No*	Hematuria, hydroureteronephrosis	LPT with left nephro-ureterectomy, pelvic LNT, proctectomy	CHT (carboplatin/ taxol)	Native left mid-ureter	EAC (grade II-III)	U, alive
**Nomoto K. et al.[31] (2010)**	57	No	G1 P1. BMI 32, fibromatosis	Pelvic endometriosis	Histerectomy and adesiolisis in pouch douglas	*No*	AVB	Total abdominal vaginectomy, pelvic LNT, left salpingo-oophorectomy	No	Vagina	EAC	12, alive
**Hirabayashi K. et al.[32] (2009)**	73	No	G3 P3	Adenomyosis	None	*No*	Slight fever, weight loss	TAH and BSO	CHT	Uterus	CCC	7, recurrence
**Efthymiou C.A. et al.[33] (2009)**	59	E+T for 13 y	None relevant	Severe ovarian and pelvic endometriosis	Umbilicus excision of endometriotic deposit. TAH and BSO	*Yes*	Constipation, tenesmus and 7 kg weight loss	Anterior rectum resection with covering loop ileostomy	No	Rectum	EAC	U, alive
**Karanjgaokar V.C. et al.[34] (2009)**	60	E for 14 y	P2	Ovarian endometriosis and adenomyosis	TAH and BSO	*Yes*	Intermittent right sided abdominal pain	Right hemicolectomy with excision of the retroperitoneal cyst	aromatase inhibitor, CHT	Retroperitoneal	Adenosarcoma and endometioid adenocarcinoma	U
	56	E+T for 12 y	G0	Endometiosis in the ovary, uterine sierosa and bowel	Laparohysterectomy and left salpingo oophorectomy; right salpingo oophorectomy with appendicotomy	*Yes*	Recent onset of discompfort in her right iliac fossa	Debulking surgery	CHT (carboplatin)	Pelvis	EAC	U
	55	E+T for 9 y	P2	Endometriosis and left ovarian endometrioma	STH and BSO	*Yes*	Cramp like discomfrot in the abdomen ant thighs and postmenopausal bleeding	Excision of the tumor mass including removal of the cervix and rectosigmoid en bloc with right hemicolectomy	CHT (ifosfamide, doxorubicin)	Pelvis	High grade endometrial stormal sarcoma	U
**Motohara K. et al.[35] (2008)**	52	No	Hypermenorrhea, dysmenorrhea, fibromatosis	Adenomyosis	No	*No*	AVB	TAH, pelvic LNT	CHT	Uterus	EAC	60, alive
**Al-Talib A. et al.[36] (2008)**	68	E	Fibromatosis	Endometriosis	TAH and BSO	*Yes*	Left-sided pelvic mass and shortness of breath	None	CHT (carboplatin/ paclitaxel)	Pelvis	Metastatic EAC	24, alive
**Noel J.C. et al.[37] (2006)**	75	Soy isoflavones for 5 y	None relevant	Extensive endometriosis and adenomyosis	TH and BSO	*Yes*	Chronic abdominal pain, left pyelonephritis	Small bowel and partial bladder resection, left nephroureterectomy	Letrozole	Right ureter	Mullerian carcinosarcoma (mixed mullerian tumor)	3, alive
**Nomura S. et al.[38] (2006)**	40	EP + CEE for 22 months	G0.	Endometriosis, adenomyosis	TAH and BSO	*Yes*	Solid mass involving the vaginal cuf	None	CHT (carboplatin/ paclitaxel – irinotecan/ carboplatin; RT	Vaginal cuff	EAC	16; deceased
**Shah C. et al.[39] (2006)**	55	No	U	No	No	*No*	AVB, polypoid vaginal lesion	TAH, BSO, upper vaginectomy, pelvic and peri-aortic LNT	No	Vagina	CCC	7, alive
**Hirabayashi K. et al.[40] (2006)**	50	No	G0	Ovarian endometrioma	Left salpingo oophorectomy	*Yes*	Abdominal pain and distention, abdominal tenderness, palpable pelvic mass	TAH, right salpingo oophorectomy, LNT	CHT	Ovary	EAC, small cells carcinoma, choriocarcinoma, CCC	10, deceased
**Milam M.R. et al.[41] (2006)**	47	E for 16 y	None relevant	Left and right endometriomas	Surgical menopause	*Yes*	Persistent and enlarging right groin mass, right lower quadrant tenderness	Surgical en bloc excision	No	Inguinal	Adenosarcoma	12, alive
**Taylor A. et al.[42] (2005)**	55	U	Fibromatosis	Ovarian endometriosis	TAH and BSO; excision of a vault endometrioma	*Yes*	ABV and dyschezia	LPT: inoperable frozen pelvis	No	Pelvi, vagina	EAC	U
	54	No	None relevant	Ovarian endometriosis	LPT excision of ovarian endometrioma	*Yes*	Pain and an abdominal mass	TAH, BSO, omentectomy	No	Ovary and uterine fundus	Moderately differentiated EAC	U
**Chang H.Y. et al.[43] (2005)**	37	CEE for 11 y	G0 P0	Extensive endometriosis (ovarian)	TAH and BSO	*Yes*	Vaginal bleeding	Cytoreductive surgery	No	Cul de sac, peritoneum	Adenosarcoma	U
**Somoye G.O. et al.[44] (2005)**	68	E for 20 y	Thyrotoxicosis.	Endometriosis.	TAH and BSO	*Yes*	Pelvic discomfort and hematuria	None	RT	Vagina and bladder	Adenocarcinoma	U
**Kawate S. et al.[45] (2005)**	62	CEE for 14 y	Fibromatosis	Deeply infiltrating endometriosis	TAH and BSO	*Yes*	Abdominal mass	Sigmoidectomy with LNT	CHT	Sigmoid colon	EAC	28, alivee
**Salerno M.G. et al.[46] (2005)**	54	Estradiol for 5 y	Fibromatosis, bilateral serous ovarin cysts	No	TAH and BSO	*No*	Pelvic pain, recurrent cystitis	Mass partially removed, lomboaortic and pelvic LNT	CHT	Right ureter	EAC with squamous differentiation	12, alive
**Jelovsek J.E. et al.[47](2004)**	52	GnRh analogues	G1 P1	Endometriosis.	TAH and BSO	*No*	Flulike symptoms, right-sided shoulder pain, and pain with inspiration	Resection of the suspected liver endometrioma and pelvic LPTwith peritoneal biopsies	No	Liver	Mullerian adenosarcoma	U, alive
**Lacroix – Triki M. et al.[48](2004)**	45	GnRh analogues	None relevant	Aggressive neurological endometriosis	None	*No*	Disabling sciatica with motor deficit	TAH, BSO, excision of pelvic endometriotic pockets	CHT, GnRH-a, palliative RT	Left pelvis (sciatic nerve)	Low grade endometrial stromal sarcoma	Deceased
**Soliman N.F. et al.[49] (2004)**	60	CEE for 3 y	Microinvasive squamous carcinoma of the cervix	Pelvic endometriosis	TAH and BSO	*No*	Heavy, painless vagin bleeding, palpable fixed mass, polypoid necrotic lesion	LPT with partial mass excision	RT	Pelvis	Moderatly differentiated EAC	U, alive
	51	E+T for 10 y	None relevant	Extensive endometriosis, endometriotic right ovarian cyst	TAH and right salpingo oophorectomy	*Yes*	Painless mass in the right side of the abdomen and several episodes of vaginal bleeding	Excision of the mass, vaginal vault, right nephro-ureterectomy and left salpingo-oophorectomy	No	Pelvis	EAC	U, alive

**Garvan F. et al.[50] (2004)**	53	No	None relevant	No	other	*No*	Intermittent painful gross haematuria	Hysterectomy with resection of the posterior wall of the bladder	No	Bladder	EAC	U
**Areia A. et al.[51] (2004)**	53	E for 6 months	Fibromatosis	Endometriotic foci especially in Fallopian tubes	TAH and BSO	*No*	AVB	Debulking surgery	CHT	Vaginal vault, bladder, rectosigmoid	EAC	6, alive
**Bese T. et al.[52] (2003)**	74	No	G3 P1. Hyperthyroidism, hypertension. BMI 29.7. Breast carcer. Tamoxifen for 2 years	No	STH and BSO. Modified righ radical mastectomy	*No*	AVB	Resection of cervix, rectum, and the accompanying mass	RT	Cervix uteri and rectal muscular wall	Adenocarcinoma	22, alive
**Okugawa K. et al.[53] (2002)**	67	No	G0. Breast carcinoma. Tamoxifen citrate for 4 y	No	Hysterectomy + BSO	*No*	Genital discharge	TAH, BSO, pelvic LND dissection, paraaortic lymph node biopsy	CHT	Adnexa	EAC	48, alive
**Petersen V.C. et al.[54] (2002)**	61	Estrogen for 5 y	None relevant	Endometriosis	Surgical menopause	*Yes*	Diarrhoea, right buttock pain, rectal mass	Anterior resection with formation of a loop Ileostomy	No	Large intestine (colorectal)	Poorly differentiated EAC	28, alive
	57	8 years	None relevant	Ovarian endometriosis	Surgical menopause	*No*	Lower abdominal pain, rectal mass	Anterior resection	No	Rectum	Variably differentiated EAC	18, alive
**Jones K.D. et al.[55] (2002)**	52	Estardiol for 12 y	G0	Extensive deeply infiltrating rectovaginal endometriosis	TAH and BSO	*Yes*	Rectal bleeding and polyp arising from sigmoid colon	Anterior resection of the rectosigmoid colon	No	Distal sigmoid colon	Well differentiated EAC	9, alive
**Powell J.L. et al.[56] (2001)**	56	EP for 3 y, E for 7 y	Fibromatosis	Extensive endometriosis, left ovarin endometrioma, adenomyosis	TAH and BSO	*Yes*	lower abdominal pain, dyspareunea, hirsutism, pain with bowel movements	Surgery	CHT; progestin therapy	Rectovaginal septum, vault	EAC	9, alive
**Lavery S. et al.[57] (2001)**	50	E for 13 y	Urogenital prolapse	left endometriotic cyst	Vaginal hysterectomy + BSO	*No*	Pain, dyspareunea, urinary frequency. Small low cystocoele and a small rectocoele	Rsection of the mass, resection of sigmoid colon and a reanastomosis; bladder opening and raise of Boari flap with reanastomosis of ureter over a ureteric stent.	No	Vaginal vault	Adenocarcinoma	U
	53	E for 3 y	None relevant	Ovarian endometriosis, adenomyosis	TAH and BSO	*Yes*	Abdominal discomfrot, swelling, diarrhea, ascites, slightly distended abdomen, firm mass at the vault of the vagina	LPT and biopses	No	Peritoneal cavity, bowel, vagina	EAC +CCC	U
**Debus G.[58] (2001)**	50	E	G0	Severe endometriosis	TAH and BSO	*Yes*	Pelvic mass	LPT with excision of the pelvic mass and rectosigmoid colon, pelvic and paraaortic LNT	CHT	Pelvic mass	EAC	U, alive
**Magtibay P.M. et al.[59] (2001)**	76	U	Breast cancer	Adenomyosis, endometriosis	TAH and BSO. Resection of the mass and of the upper portion of the vagina	*No*	Diarrhea, bright red blood per rectum	LPT with distal transection of the rectum	RT	Rectum, vagina	EAC (grade II)	21, alive
**Jimenez R.E. et al.[60] (2000)**	48	CEE for 5 y	Fibromatosis	Endometriotic ovarian cyst	TAH and BSO	*No*	Unilateral hydronephrosis	LPT dissection of the mass, right nephroureterectomy, partial colectomy and partial small bowel resection	No	Ureters	Adenosquamous ednometrioid carcinoma	U
**Mc Cluggage W.G. et al.[61] (2000)**	50	No	G0. Breast cancer. TMX for 3 years	No	No	*No*	AVB, bulky uterus	TAH and BSO	No	Right ovary and uterus	EAC	U
**Jennings T.S. et al [62](1999)**	70	No	Infertility. Breast cancer trated with Tamoxifen	No	Right modified radical mastectomy	*No*	Abdominal pain, abdominal mass, constipation	TAH, BSO, infracolic omentectomy and pelvic washing	CHT	Ovary	CCC	2, alive
**Schlesinger C. et al.[63] (1999)**	62	No	G0. Obesity, hypertension, fibromatosis, breast cancer. TMX for 2 years	No	Left partial mastectomy and lymphadenectomy othrt	*No*	AVB, endometrial polyp	TAH and BSO	No	Uterus	EAC endometrioid type	U
**Taylor M. et al.[64] (1999)**	42	E for 7 y	None relevant	Severe endometriosis	TAH and BSO	*Yes*	Massive ascitis		CHT	Pelvis	EAC	2, alive
**Sidhu S. et al.[65] (1998)**	60	18 years (E then CEE)	G4 P4.	Extensive endometriosis	TAH nd BSO. Anterior colon resection.	*Yes*	Pain, mass	Debulking surgery (not possible complete resection)	RT	Pelvis	Sertiliform endometrioid adenocarcinoma	U
**Horiuchi A. et al.[66] (1998)**	53	No	None relevant	No	None	*No*	Pelvic mass, ascites, high fever, low abdominal pain	TAH, BSO, pelvic LNT	CHT (carboplatino/ vyncristin/ cisplatin/ actinomycin/ pepleomycin)	Left ovary	EAC, Yolk Sac Tumour arising from endometriotic cyst	6, deceased
**Abu M.A.E. et al.[67] (1997)**	38	E for 13 y	None relevant	Extensive endometriosis involvign both ovaries, adenomyosis	TAH and BSO	*No*	Intermittent vaginal bleeding. Ulcerated area over vaginal vault, polyp like lesion on vaginal vault found 4 weeks later	Unspecified surgery	Neoadjuvant progestin therapy, RT	Pelvis	EAC	U

**Eichhorn J.H. et al.[68] (1996)**	43	High-dose combination CO 3 months	None relevant	Endometriosis,	No	*No*	AVB, marked fall in hematocrit	TAH, BSO, partial colectomy	No	Ovary	Endometrioid cell-ciliated tumors (EAC)	42, alive
	44	No	None relevant	Endometriosis	No	*No*	Pelvic mass	TAH, BSO, peritoneal biopses	No	Ovary	Endometrioid cell-ciliated tumors (EAC)	21, alive
**Mc Cluggage W.G. et al.[69] (1996)**	62	E for 5 y	Simple serous cystadenoma	No	TAH and right ovarian cystectomy	*No*	AVB	Partial vaginectomy, bilateral oophorectomy and partial omentectomy	No	Left ovary, rectum, sigmoid colon, retrovaginal septum	Endometrial stromal sarcoma	3, alive
**Cohen I. et al.[70] (1994)**	71	U	Infertility, breast cancer. TMX for 1 year	No	Right radical mastectomy; LPT for treatment of bilateral tubal occlusion	*No*	Asymptomatic	TAH, BSO, periaortic lymph node sampling	CHT	Ovary	EAC	24, alive
**Dunn S. et al.[71] (1993)**	62	Estradiol for 20 y	G2 P2	Severe endometriosis (ovaries and rectovaginal septum)	TAH and BSO	*Yes*	Pelvic mass	Mass excision en bloc with rectosigmoid colon	RT	Rectosigmoid colon	EAC	1.5, deceased
**Vara A.R. et al.[72] (1990)**	62	No	AVB, fibromatosis	Cervical endometriosis	TAH and BSO 10 y previously	*No*	Gross hematuria, 20-pound weight, vague suprapubic pain	Radical cystectomy and urethrectomy with wide excision of the anterior vaginal wall	No	Bladder	Endometrioid adenosarcoma	12, alive
**Heaps J.M. et al.[73](1990)**	47	E for 7 y	None relevant	Endometriosis	TAH and BSO	*No*	Pelvic mass, bleeding, pain	U	No	Cul de sc, peritoneum	Adenocarcinoma	U
	59	E for 5 y	None relevant	Endometriosis	TAH and BSO	*No*	Pelvic mass, bleeding, pain	U	No	Cul de sac, peritoneum	Adenocarcinoma	U
	62	E for 11 y	None relevant	Endometriosis	TAH and BSO	*No*	Pelvic mass, bleeding, pain	U	No	Cul de sac, peritoneum	Adenocarcinoma	U
	47	E for 2 y	None relevant	Endometriosis	TAH and BSO	*No*	Pelvic mass, bleeding, pain	U	No	Cul de sac, peritoneum	Adenosquamous carcinoma, CCC features	U
**Orr J.W. et al.[74] (1989)**	60	E for 22 y	G0	Pelvic endometriosis	TAH and BSO	*Yes*	Pelvic mass	En bloc resection with low rectal reanastomosis, pelvic, LNT, para-aortic node biopsy	RT	Vaginal vault and parametrium	Adenocarcinoma	6, recurrence
**Al Izzi M.S. et al.[75] (1988)**	62	No	Abdominal pain and intermenstrual/ postcoital bleeding	Bladder endometriosis	AH and BSO (13 y before)	*No*	Painless haematuria	Total radical cystectomy and ileal loop diversion	No	Bladder	EAC with occasional foci of clear cell differentiation	24, alive
**Remnitz C. et al.[76] (1988)**	58	CEE for 12 y	Leiomyoma	Extensive endometriosis	TAH and BSO	*Yes*	Beeding, pain, unilateral renal obstruction	Surgery	Progestin therapy	Pelvis	EAC	60, alive
	47	CEE for 4 y	None relevant	Extensive endometriosis	TAH and BSO	*yes*	Pain, renal obstruction	Undefined surgery	Progestin therapy, RT, CHT	Pelvis	Adenocarcinoma, adenosquamous carcinoma	1, deceased
**Brunson G.L. et al.[77] (1988)**	63	DES for 4 y	G2 P2, fibromatosis	No	TAH and BSO	*No*	Pain, postprandial nausea and vomiting	Segmental ureterectomy and reimplantation	RT	Ureter	Adenocarcinoma	U, alive
	59	No	None relevant	Severe endometriosis	TAH and BSO	*Yes*	Left lower abdominal pain and gross hematuria	Left nephroureterectomy	CHT (5fluorouracil/ adryamicin/cytoxan/ magace)	Ureter	Adenocarcinoma	22, deceased
**Granai C.O. et al.[78] (1984)**	47	E for 13 y	None relevant	Endometriosis	TAH and BSO	*Yes*	AVB	U	No	Vagina	Adenocarcinoma	U
**Kapp D.S. et al.[79] (1982)**	35	CEE for 7 months	G2 P2.	Endometriosis	Cesarean section. TAH and BSO	*Yes*	AVB	No	RT	Vagina	Adenocarcinoma	7, alive
**Shamsuddin A.K.M. et al.[80] (1979)**	55	DES for 14 y	Fibromatosis	Sever endoemtriosis	TAH and BSO	*Yes*	Pain, nocturia, dysuria	Segmentary resection of sigmoid colon and left ureter, partial cystectomy and left sigmoid colostomy	CHT	Vescicovaginal septum	EAC	U
**Lott J.V. et al.[81] (1978)**	53	DES for 15 y	G3 P3	Pelvic endometriosis	TAH and BSO	*Yes*	Rectal bleeding	Anterior resection of the sigmoid and rectum	No	Rectum	EAC	U, alive
**Berkowitz R.S. et al.[82] (1978)**	57	CEE for 13 y	None relevant	Endometriosis	TAH and BSO	*Yes*	AVB	U	No	Vagina	Endometrial stromal sarcoma	U
**Brooks J.J. et al.[83] (1977)**	48	E for 4 y	G0, fibromatosis	Extensive endometriosis, adenomyosis	STH and BSO	*Yes*	Mild right sided lower abdominal pain, urinary frequency, constipation	Unspecified surgery	RT	Retroperitoneal	CCC	22, alive
**Youg E. et al.[84] (1969)**	47	CEE + T for 14 y	None relevant	No	TAH and BSO	*No*	AVB	Total pelvic exenteration	No	Rectovaginal	Adenoacanthoma	U, alive

Abbreviations: TLH: total laparoscopic hysterectomy; BSO: bilateral salpingo oophorectomy; TAH: total abdominal hysterectomy; RAH: radical abdominal hysterectomy; STH: subtotal hysterectomy; AH: abdominal hysterectomy; D&C: dilatation and currettagre; HSC: hysteroscopy RT: radiotherapy; CHT: chemioterapy; LPS: laparoscopy; LPT: laparotomy; MRI: magnetic resosance; CT: computed tomography; US: ultrasound; Y: years; G: gravid; P: parity; U: unspecified; EOC: epithelial ovarian cancer; CCC: clear cell carcinoma; EAC: endometrioid adenocarcinoma; M: metastases; HRT: hormone replacement therapy; CEE: conjugated equin estrogen; U: unspecified; AVB: abnormal vaginal bleeding; LNT: lymphoadenectomy; DES: diethylsilbestrol; T: testosterone.
